# Efficacy and safety of metronomic oral vinorelbine in elderly patients with locally advanced and metastatic non-small cell lung cancer

**DOI:** 10.1186/s12885-026-16517-x

**Published:** 2026-07-17

**Authors:** Sharehan Hassan Soliman, Mahinour Mohamed Atef

**Affiliations:** Clinical Oncology and Nuclear Medicine Department, Faculty of Medicine, Suez Canal University, Ismailia, Egypt

**Keywords:** Vinorelbine, Elderly patients, Non-small cell lung cancer

## Abstract

Lung cancer remains one of the most frequently diagnosed malignancies and the leading cause of cancer-related mortality globally. Metronomic chemotherapy (MC) enables prolonged low-dose treatment administration with lower toxicity while maintaining therapeutic efficacy. Our goal was to assess the efficacy and safety of oral vinorelbine (OV) administered in a metronomic schedule in elderly patients with locally advanced or metastatic non-small cell lung cancer (NSCLC).

**Patients & methods**

This single-center study was conducted at the Clinical Oncology Department of Suez Canal University Hospital. The study included elderly patients (aged ≥ 60 years) with locally advanced or metastatic NSCLC (TNM stage IIIB/IV) who had previously received first-line platinum-based chemotherapy with or without concomitant chemoradiotherapy. All patients were confirmed to lack sensitizing EGFR mutations as well as ALK and ROS1 rearrangements, irrespective of PD-L1 expression status and were treated between January 2020 and October 2024. The study population consisted of histopathologically-proven patients who fulfilled the inclusion and exclusion criteria. Patients received metronomic OV at fixed doses (30, 40, or 50 mg three times weekly) until disease progression or development of unacceptable toxicity.

**Results**

From January 2020 to October 2024, 50 patients were included: 39 men (78%) and 11 women (11%). The median age was 67 years. The predominant histological subtype was squamous cell carcinoma (44%). The duration of OV therapy ranged from 6 to 18 months, with a mean of 9.16 ± 3.6 months. Patients received 8 to 24 cycles, with a mean of 11.74 ± 4.44. According to RECIST criteria, the disease control rate was 78% after 6 months, while the overall response rate was 64%. No grade 4 or 5 adverse events were observed. The mean progression-free survival (PFS) was 13.74 months (95% CI: 12.11 to 15.36), whereas the mean overall survival (OS) was 19.67 months (95% CI: 17.85 to 21.49). Comparison of EORTC QLQ-C30 quality-of-life (QoL) scores before treatment and after 6 months of OV therapy demonstrated improvement in patients' functioning and QoL.

**Conclusion**

Metronomic OV demonstrated clinical efficacy in elderly patients with advanced wild-type NSCLC. It was associated with a significant reduction in toxicity and may represent a valuable therapeutic option for this age group, with excellent treatment compliance.

**Supplementary Information:**

The online version contains supplementary material available at 10.1186/s12885-026-16517-x.

## Introduction and background

Non-small cell lung cancer (NSCLC) remains the leading cause of cancer-related mortality in Western countries [1]. In Egypt, lung cancer is among the most lethal malignancies; nonetheless, it is the fourth most prevalent cancer among males and relatively uncommon in females. The gender disparity in incidence is largely attributable to differences in tobacco smoking rates [2].

According to Giovino et al., Egypt continues to report one of the lowest smoking rates among women worldwide. Egypt does not have a nationwide screening program; hence, most patients have locally advanced or metastatic disease [3].

Most diagnoses occur at advanced stages, and existing therapies remain limited in effectiveness. Individuals over the age of 65 account for more than half of advanced NSCLC cases, while those over the age of 70 represent approximately 30% to 40% [4].

Because of their unique circumstances, elderly patients must carefully weigh the risks and benefits of therapy. Selecting the optimal treatment for patients with medical comorbidities and social challenges can be difficult [5]. Many elderly patients with advanced NSCLC benefit from single-agent chemotherapy [6].

Both intravenous vinorelbine and oral vinorelbine (OV) are routinely used and have favourable toxicity profiles, making them appropriate options for the elderly and/or frail patients. Metronomic chemotherapy (MC) allows for the administration of higher drug doses while maintaining safety. The recommended approach involves administering single fixed pharmaceutical doses at regular intervals until disease progression or severe toxicity [7, 8].

MC is a non-cytotoxic therapeutic strategy that aims to overcome drug resistance. Metronomic administration of chemotherapy leads to a cytostatic effect by shifting the treatment target from cancer cells to tumor angiogenesis. Shifting the therapeutic target from tumor cells to tumor vasculature may inhibit tumor regrowth between treatment cycles [9].

In three-phase I studies, oral metronomic VNR was investigated at a reference dose of 50 mg administered three times per week (Monday, Wednesday, and Friday). The trials revealed that the regimen was both safe and effective. Furthermore, Briasoulis et al. (2009) reported similar findings [10].

This study aims to improve the quality of care for elderly patients with locally advanced or metastatic NSCLC using different therapeutic approaches. The primary endpoints were to assess the efficacy and safety profile of OV as MC in elderly patients with locally advanced or metastatic NSCLC. The secondary endpoints were to evaluate the progression-free survival (PFS) and overall survival (OS) of elderly patients with locally advanced and metastatic NSCLC receiving metronomic OV.

## Patients & methods

### Research design

This was a single-center retrospective cohort study. Data were collected from a prospectively maintained database at the Clinical Oncology Department, Suez Canal University Hospital. Our practice team routinely performed pre- and post-treatment assessments for elderly patients with locally advanced and metastatic NSCLC patients who received metronomic OV according to the departmental policy, as mentioned by previously published data [11–17]. These data were extracted and collected from medical records to evaluate the efficacy and safety profile of metronomic OV in elderly patients, as well as patient quality of life. Analysis of the collected data was retrieved.

### Study setting

The study was carried out at Clinical Oncology and Nuclear Medicine Department, Suez Canal University Hospital, Ismailia, Egypt. Patients with locally advanced or metastatic NSCLC (TNM stage IIIB/IV) who had received first-line platinum-based chemotherapy and/or concomitant chemoradiotherapy were enrolled to receive metronomic OV according to the predefined inclusion and exclusion criteria. Patients who fulfilled the inclusion criteria were included in the study.

### Patient selection and data collection

The study included elderly patients diagnosed with locally advanced or metastatic NSCLC between January 2020 and October 2024.

### Inclusion criteria

1. Age ≥ 60 years with locally advanced or metastatic NSCLC (TNM stage IIIB/IV) who previously received first-line platinum-based chemotherapy and/or concomitant chemoradiotherapy and are not amenable to surgery. According to the Central Agency for Public Mobilization and Statistics (CAPMAS) and the Egyptian census, “older persons” or “elderly” are defined as individuals aged 60 years or older.
2. Patients with cytologically or histologically confirmed locally advanced or metastatic (stage IIIB/IV) NSCLC (TNM 7th edition 2009) with RECIST 1.1 measurable disease at diagnosis.
3. Patients without oncogenic driver mutations, including EGFR mutations, ALK translocations, or ROS1 rearrangements. PD-L1 expression level could be either positive or negative.
4. ECOG performance status (PS) 0–2, with a life expectancy greater than 6 months.
5. Adequate hepatic, renal, cardiac and bone marrow function.

Criteria for adequate organ function included: bone marrow function (neutrophils ≥ 2*10^9^/I, platelets ≥ 100*10^9^/I, hemoglobin ≥ 10.0 g/dL); hepatic function (total bilirubin < 1.5*ULN, transaminases [ALT, AST] < 2.5*ULN, alkaline phosphatase < 5*ULN); and renal functions (calculated creatinine clearance ≥ 30 ml/min (Cockcroft and Gault formula).

### Exclusion criteria

1. Patients with symptomatic brain metastases or Severe cognitive disorders.
2. Patients aged < 60 years.
3. Inability to swallow oral medication.
4. History of previous or concurrent malignancy.
5. Presence of EGFR, ALK, or ROS1 mutations.
6. Severe comorbidities, such as heart disease (previous myocardial infarction, heart failure, valvular heart disease, or serious arrhythmias), chronic obstructive pulmonary disease (COPD), cerebrovascular or peripheral vascular disease, chronic renal failure, hepatitis and/or cirrhosis, severe autoimmune diseases, or Symptomatic ascites or pericardial effusion.
7. Patients with large cell neuroendocrine cancer

### Sample size

The sample size was determined using the following equation [18]:

$$\mathbf{n}=\frac{(\mathbf{Z}_{\boldsymbol{1}-\frac{\boldsymbol{\upalpha}}{\boldsymbol{2}}})^{\boldsymbol{2}} * \mathbf{p} (\boldsymbol{1}-\mathbf{p})}{\mathbf{d}^{\boldsymbol{2}}}$$

Where:

- *n* = sample size
- Z_1_-α/2 = the confidence interval, which equals 1.96 when the type 1 error is 5%
- *P =* prevalence of non-small cell lung cancer, estimated at 85% of all lung cancer [19, 20]
- d = Absolute error or precision, usually equals 10%

The calculated sample size, after accounting for expected (drop-out) rate of 10%, was 50 participants.

**Fig. 1:**
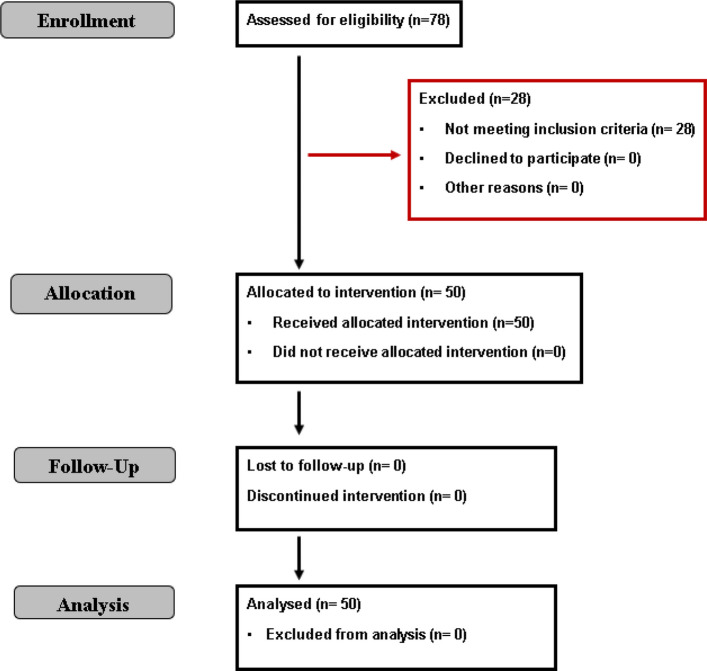
Flow chart showing patient inclusion, exclusion, and participation throughout the study

A total of 78 patients were initially identified and screened from the Suez Canal University Hospital cancer registry during the period from January 2020 to October 2024. Following application of the predefined inclusion and exclusion criteria, 50 patients met the eligibility criteria and were subsequently included in the study. To enhance transparency and clarify the selection process, Fig. 1 illustrates the patient screening and enrollment flow diagram.

During the initial screening, 28 patients were excluded for failing to meet the predefined eligibility criteria, including age < 60 years or ECOG performance status > 2. Following the enrollment of the 50 eligible patients, no additional exclusions occurred, and there was no loss to follow-up among the included participants during the study period.

A comprehensive sampling (total population) approach was adopted. Based on prior power analysis, a minimum sample size of 50 patients was considered sufficient. A systematic review of the Suez Canal University Hospital cancer registry for the period from January 2020 to October 2024 identified exactly 50 patients who fulfilled all predefined inclusion and exclusion criteria. Accordingly, all eligible patients were consecutively enrolled, representing the entire accessible population.

### Treatment protocol

Baseline assessment included a detailed medical history, physical examination, assessment of clinical presentation and patient performance status.

A history of chronic illnesses (defined as a condition lasting one year or more that requires continuous medical treatment or limits daily activities, or both, according to the Centers for Disease Control and Prevention (CDC) definition, www.cdc.gov) was recorded.

Enrolled patients had locally advanced or metastatic NSCLC (TNM stage IIIB/IV) and had received first-line platinum-based chemotherapy and/or concomitant chemoradiotherapy, identified through the department computer system. Baseline staging included brain Magnetic Resonance Imaging (MRI) and Spiral Computed Tomography (CT) scans.

Health-related quality of life (HRQoL) was assessed before enrollment and after 6 months of metronomic OV using the EORTC QLQ-C30 questionnaire.

Oral vinorelbine (OV) was administered at doses of 50, 40, or 30 mg three times per week (Saturday, Monday, Wednesday) continuously until disease progression or unacceptable toxicity. Patients received OV on days 1, 3, and 5 of each week, according to their tolerance, comprising a 21-day cycle. Treatment continued until the disease progression, intolerable toxicity, patient refusal, or investigator decision.

Vinorelbine capsules were taken after a meal without chewing or sucking them. No primary antiemetic prophylaxis was recommended, but was delivered upon request. In case of diarrhea, loperamide was recommended. Granulocyte colony-stimulating factors were permitted in grade 3 neutropenia with fever lasting ≥ 3 days or in case of grade 4 neutropenia. Erythropoietin use was allowed. Patients received treatment at home and were seen every cycle, with complete blood counts and serum chemistry being performed before each cycle, which was repeated every 21 days.

#### Metronomic oral vinorelbine protocol

The starting dose of oral vinorelbine was 50 mg (one 30 mg plus one 20 mg capsule), administered after lunch on a continuous thrice-weekly schedule (Saturday, Monday, Wednesday) until disease progression or unacceptable toxicity occured.

Dosing does not inherently change based on the line of therapy, but is instead determined by patient-specific factors such as ECOG performance status and age, with 30,40,50 mg administered three times weekly.

#### Dose modification and toxicity management

- *General Toxicity*: For grade 3 toxicity or grade 2 toxicity significantly affecting daily activities, the dose was reduced to 40 mg. If toxicity persists or further deterioration occured, the dose was reduced to 30 mg. Treatment was permanently discontinued for severe, unmanageable toxicity.
- *Hematological Toxicity*: For grade 3–4 hematological toxicity or persistent grade 2 toxicity lasting over three weeks with clinical impact, the dose was reduced to 30 mg three times per week.
- *Non-Hematological Toxicity*: Treatment was delayed or interrupted in cases of persistent grade 2 toxicity affecting daily life or grade 3 diarrhea.
- *Management of Adverse Events (AEs)*: Any other grade 3 or 4 AE led to treatment delay for up to three weeks; If toxicity persisted, treatment was permanently stopped.

### Primary and secondary endpoints analysis

The primary outcome was assessment of efficacy and safety (adverse events). Key secondary endpoints included achieving a high disease control rate (complete response [CR] + partial response [PR] + stable disease [SD]), a high objective response rate (CR + PR), PFS, OS, and better quality of life (QoL).

- *PFS* was calculated from the start of metronomic OV to the date of progression.
- *Overall response rate* (ORR) was defined as the best response (CR and PR) per RECIST criteria after 6 months of OV.
- *Overall survival (*OS) was measured from the start of metronomic OV until death or the cutoff date of October 2024. For patients who had not died, the survival time was measured up to the cutoff date. All deaths were considered events regardless of cause, including deaths unrelated to cancer, providing a pragmatic, real-world estimate of survival outcomes in this elderly cohort.

With respect to censoring, no patients were lost to follow-up during the study period. Accordingly, all censoring was purely administrative and occurred only for patients who were confirmed to be alive at the predefined study cutoff date (October 2024).

No data imputation was required due to the 100% follow-up rate and the complete survival data for all participants; the Kaplan–Meier survival estimates accurately reflect the observed clinical outcomes of the entire study population without the need for statistical handling of missing values. Follow-up visit delays were managed via phone calls. Assessment of measurable disease was conducted and evaluated after 6 months using the RECIST criteria with spiral computed tomography (CT) scanning and brain magnetic resonance imaging (MRI) performed. All radiological evaluations were conducted in accordance with RECIST 1.1 Criteria. To enhance objectivity and minimize assessment bias, two senior radiologists independently reviewed radiologic studies. In instances of discrepancy regarding treatment response, a third senior consultant radiologist was consulted to adjudicate and reach a final consensus decision.

Safety was assessed on the first day of each cycle with physical examination, vital signs, body weight, performance status (PS), complete blood counts, serum biochemistry, and the QoL questionnaire (EORTC QLQ C30). Safety assessment was rated using the NCI-CTCAE classification version 6.0. Adverse events were recorded for each cycle and patient. All safety analyses were conducted independently of treatment connection or relatedness. All significant adverse events (SAEs) and treatment-related SAEs were recorded and tabulated.

QoL was assessed using the EORTC QLQ C30 immediately before and after 6 months of OV therapy. The QLQ-C30 included five functional scales, three symptom scales, a global health status/QoL scale, and six single items scored 0–100. Higher scores indicate a better reaction level.

The EORTC QLQ-C30 questionnaire was administered to all eligible participants during follow-up. All 50 patients (100%) completed the assessment, so no data imputation was required. To minimize potential recall bias, patients were instructed to anchor their responses to clearly defined clinical milestones documented in their medical records, thereby enhancing the accuracy and contextual reliability of their self-reported outcomes.

Data were extracted from the prospectively maintained at the Clinical Oncology Department database at Suez Canal University Hospital. As part of routine departmental policy, elderly patients with locally advanced or metastatic non-small cell lung cancer (NSCLC) and an ECOG performance status (PS) of 0–2 receive metronomic oral vinorelbine (OV) following completion of first-line platinum-based chemotherapy. Standard clinical practice included baseline assessment using the EORTC QLQ-C30 prior to initiation of metronomic OV. After six months of treatment, patients undergo radiological evaluation to assess treatment response, in addition to a follow-up EORTC QLQ-C30 assessment.

For this single-center study, relevant clinical, safety, efficacy, and quality-of-life data were systematically extracted from medical records and analyzed to evaluate the therapeutic outcomes and tolerability profile of metronomic OV in this elderly patient population.

### Ethical considerations

The research ethics committee of the Faculty of Medicine, Suez Canal University (FOMSCU) approved the study protocol. Clinical data were obtained with the FOMSCU study ethics committee approval. All research data and treatment procedures complied with relevant guidelines and regulations.

### Statistical analysis

Statistical analysis was performed using SPSS for Windows, version 28 (IBM Co., Armonk, NY, USA). Numerical data are presented as mean ± standard deviation (SD) and compared using paired t-test at both time periods. Categorical data are reported as frequencies and percentages.

Survival analysis used Kaplan–Meier curves with log-rank test. Cox regression analysis was employed to examine various survival variables. Linear regression analysis was used to determine the parameters related to the EORTC QLQ-C30 score after therapy. A two-tailed *P*-value < 0.05 indicated statistical significance.

## Results

### *Baseline patient characteristics

From January 2020 to October 2024, 50 patients were enrolled in the study: 39 males (78%) and 11 females (11%). The study was conducted on locally advanced and metastatic non-small cell lung cancer elderly patients (stage IIIB/IV NSCLC TNM 7th edition 2009), 36% of whom were within the age range of 60–64 years, and 42% were within the age range of 65–69 years (median age 67 years, IQR 5.5). Also, 40% were smokers, 62% had no chronic illness, while 26% were hypertensive and 12% were diabetic. This is shown in Table 1.

**Table 1:** Baseline characteristics, Clinical & pathological data of the patients studied (*n* = 50)

Item	N	%
Age group (years)		
60–64	18	36.0
65–69	21	42.0
70–74	5	10.0
75–79	4	8.0
≥ 80	2	4.0
Sex		
Male	39	78.0
Female	11	22.0
Smoking	20	40.0
Comorbidities		
No chronic illness	31	62.0
DM	6	12.0
HTN	13	26.0
Cardiac	5	10.0
Bronchial asthma	2	4.0
Presentation		
Hemoptysis	12	24.0
Weight loss	7	14.0
Dyspnea	31	62.0
Cough	29	58.0
Easy fatigability	2	4.0
Fever & night sweat	1	2.0
Pleural effusion	2	4.0
Pathology		
Undifferentiated large cell NSCLC	8	16.0
Squamous cell carcinoma	22	44.0
Adenocarcinoma	20	40.0
Stage		
3B	24	48.0
3C	8	16.0
4A	11	22.0
4B	7	14.0
ECOG		
PS1	23	46.0
PS2	27	54.0

Regarding clinical presentations, over half of the patients presented with dyspnea (62%) and cough (58%). Pathology results revealed squamous cell carcinoma, adenocarcinoma, and undifferentiated large cell NSCLC in 44%, 40%, and 16% of patients, respectively. Stage 3B tumors were the predominant stage (48%), followed by stage 4 A (22%). Furthermore, 46% were ECOG PS1 at the time of presentation, and 54% were PS2, as shown in Table 1.

### *Treatment of the studied patients

As demonstrated in Table 2, 44% of patients received first-line treatment with weekly paclitaxel and carboplatin (4–6 cycles), 16% received pemetrexed and carboplatin (4–6 cycles), and only 4% received gemcitabine and carboplatin (4–6 cycles). Additionally, 48% received concomitant chemoradiotherapy to the lung mass (total 66 GY/33# with weekly cisplatin or carboplatin).

**Table 2:** Treatment of the studied patients (*n* = 50)

Item	N	%
1^st^ line chemotherapy		
Did not receive chemotherapy	18	36.0
Received paclitaxel carboplatin weekly (4–6 cycles)	22	44.0
Received Pemetrexed carboplatin (4–6 cycles)	8	16.0
Received Gemcitabine carboplatin (4–6 cycles)	2	4.0
Concomitant chemoradiotherapy over lung mass (total 66 GY/33 with cisplatin/carboplatin)		
Received	24	48.0
Did not receive	26	52.0
PDL1		
Negative	38	76.0
Positive	12	24.0
VNR dose		
30 mg 3 times weekly	27	54.0
40 mg 3 times weekly	16	32.0
50 mg 3 times weekly	7	14.0
Treatment duration (months)		
Mean ± SD	9.16 ± 3.6	
Range	6–18	
Total number of cycles administered		
Mean ± SD	11.74 ± 4.44	
Range	8–24	

Regarding mutational analysis, all patients were negative for EGFR, ALK, and ROS1 mutations. The prevalence of PD-L1 overexpression (over 50%) was 24%.

### *Dose administration

OV was administered at a dose of 30, 40, and 50 mg three times weekly, continuously. Patients received OV on days 1, 3, and 5 of each week continuously according to patient tolerability. Oral vinorelbine is typically initiated on a regular weekly schedule, often on alternating days to allow for recovery. This continuous regimen is maintained unless the disease progresses or the patient experiences intolerable adverse effects.

Dose adjustments are implemented based on the patient's tolerance and observed toxicities:

- *First Reduction*: If a patient develops grade 3 toxicity or grade 2 toxicity that significantly interferes with daily life, the dose is reduced.
- *Second Reduction*: If another grade 3 toxicity occurred or daily functioning continued to decline, the dosage was further reduced.
- *Discontinuation*: If severe clinical deterioration occurred, treatment was permanently stopped.

The VNR doses were 30, 40, and 50 mg, three times weekly, among 54%, 32%, and 14% of the study population, respectively. The duration of treatment ranged between 6 and 18 months, with a mean of 9.16 ± 3.6 months, and patients received 8 to 24 cycles with a mean of 11.74 ± 4.44. This is shown in Table 2.

Most patients received 90% to 100% of the planned dose. Dose modifications were more frequent in the 50 mg three-weekly dose schedule. The main reason for dose modification was the occurrence of an AE & patient tolerability.

### *Assessment of treatment response

According to RECIST criteria, after 6 months of OV, 12% achieved complete response, 52% achieved partial response, 14% achieved stable disease, and 22% of patients experienced progressive disease. The disease control rate was 78%, and the overall response rate was 64%. The most frequently observed response was the partial response (52%), followed by progressive disease (22%), stable disease (14%), and complete response (12%), This is shown in Table S 1.

### *Adverse events among the studied patients

Hematological/non-hematological adverse events.

Concerning hematological adverse events, 60% developed grade 1 and 2 anemia, and 50% developed neutropenia (grade 1 and 2 in 36% and grade 3 in 14%). The most commonly observed non-hematological adverse events: 72% experienced easy fatigability, 64% experienced decreased appetite, 50% experienced abdominal pain/spasm, 48% had arthralgia, 32% had constipation, 32% had renal impairment, and 30% developed neuropathy. This is shown in Table 3.

**Table 3:** Adverse events among the studied patients (*n* = 50)

Item	N	%
Haematological toxicities		
Neutropenia		
No	25	50.0
Grade 1 and 2	18	36.0
Grade 3	7	14.0
Anaemia		
No	20	40.0
Grade 1 and 2	30	60.0
Thrombocytopenia		
No	44	88.0
Grade 1 and 2	6	12.0
Febrile neutropenia		
No	45	90.0
Grade 1 and 2	3	6.0
Grade 3	2	4.0
Non-haematological toxicities		
Nausea and vomiting	7	14.0
Easy fatigability	36	72.0
Abdominal pain/spasm	25	50.0
Decreased appetite	32	64.0
Headache	12	24.0
Upper respiratory tract infection	4	8.0
Alopecia/hair loss	8	16.0
Elevated liver enzymes		
No	40	80.0
Elevated (< threefold normal level)	8	16.0
Elevated (3-to-fivefold normal level)	2	4.0
Constipation	16	32.0
Arthralgia	24	48.0
Elevated s. creat (renal impairment)	16	32.0
Dizziness/pruritus	5	10.0
Interstitial lung disease/pneumonia	6	12.0
Hepatobiliary toxic events (elevated bilirubin)	2	4.0
Neuropathy	15	30.0
Pulmonary embolism	2	4.0
Diarrhoea	7	14.0
Weight loss	4	8.0
Mild skin rash	5	10.0
Electrolyte imbalance	2	4.0
Urinary tract infection	1	2.0
Dehydration	1	2.0
Oral fungal infection	1	2.0
Pleural and pericardial effusion	3	6.0

### *Progression-free survival analysis

**Fig. 2:**
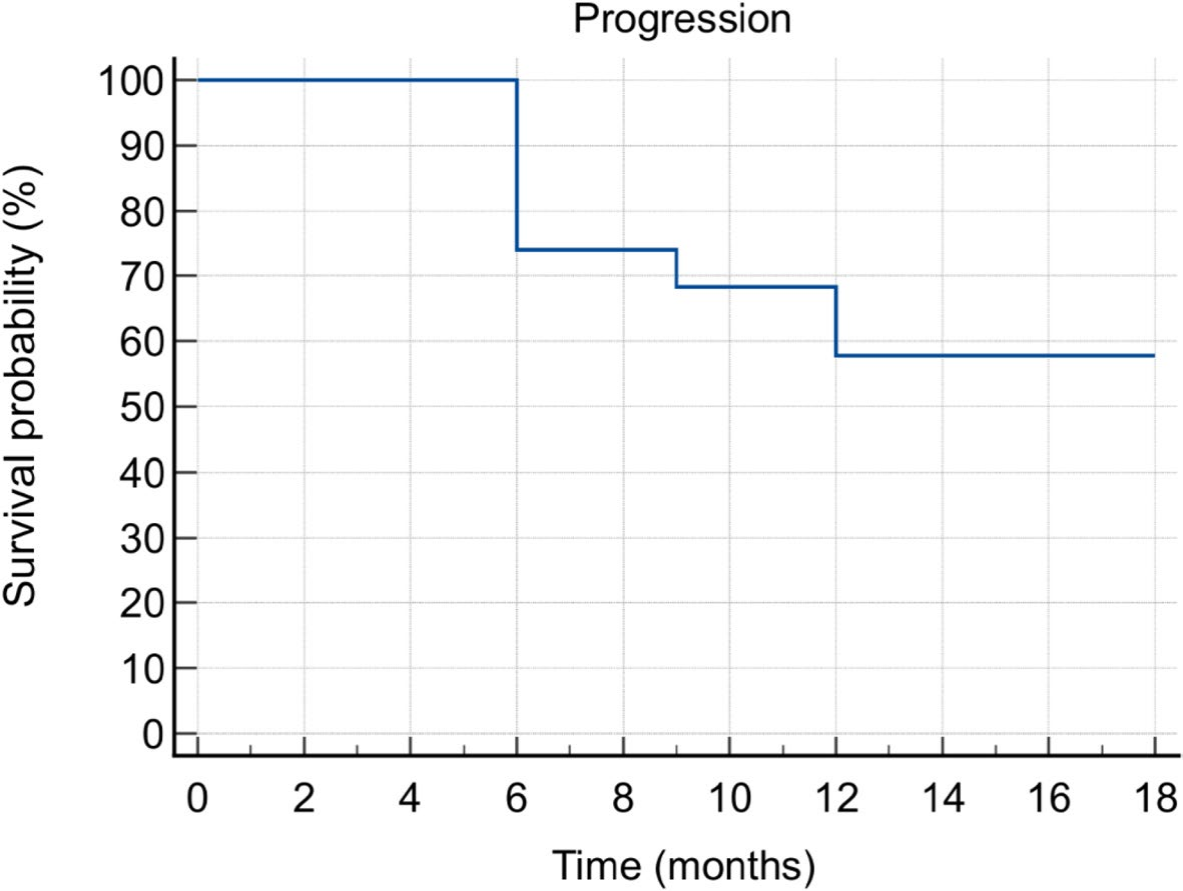
Kaplan Meier curve for PFS analysis of patients

According to Kaplan–Meier curve analysis, the studied patients remained free from disease progression for a mean duration of 13.74 months (95% CI: 12.11 to 15.36), with a progression rate of 34% Table 4, Fig. 2.

**Table 4:** Progression free survival analysis of the patients studied

Item		N of events (%)	N censored (%)		Mean survival (95%CI)	
Total patients (***n*** = 50)	17 (34%)		33 (66%)		13.74 (12.11 to 15.36)	
**Item**	**N of events (%)**		**N censored (%)**	**Mean survival**	**HR (95%CI)**	**Log-rank** ***P*-value**
Complete response	0 (0%)		6 (100%)	18		
Partial response	3 (11.5%)		23 (88.5%)	16.39	Ref	**< 0.001**
Stable disease	3 (42.9%)		4 (57.1%)	11.14	3.58 (0.88 to 14.49)	
Progressive disease	11 (100%)		0 (0%)	6	11.53 (3.05 to 43.61)

Notably, 34% of patients discontinued treatment because of progression, while 66% were still on treatment at the date cutoff date. Our investigation detected a statistically significant association between response to treatment and progression-free survival according to log-rank analysis (*P <* 0.001). Progression rates of 0% among patients who achieved complete response, 11.5% among those who showed partial response, 42.9% among those with stable disease, and 100% among those with progressive disease. The hazard was higher in the stable disease and progressive disease groups than in the partial response group, with HR (95% CI) of 3.58 (0.88 to 14.49) and 11.53 (3.05 to 43.61), respectively Figure S 1.

In univariate Cox regression analysis, tumor stage and receiving concomitant chemoradiotherapy to the lung mass were significantly associated with PFS. Patients with stage 4B tumors showed a significantly higher hazard of progression than those with 3B tumors (HR = 6.53, 95% CI: 1.63 to 26.08, *P =* 0.008), and patients receiving concomitant chemoradiotherapy showed a lower hazard than those not receiving it (HR = 0.2, 95% CI: 0.06 to 0.7, *P =* 0.012).

In multivariable analysis, tumor stage and ECOG performance status were significantly associated with PFS, as patients with stage 4B tumors showed a significantly higher hazard of progression than those with stage 3B tumors (HR = 20.31, 95% CI: 1.46 to 282.04, *P =* 0.025), and patients with ECOG PS2 had a higher hazard than those with PS1 (HR = 8.42, 95% CI: 1.11 to 63.81, *P =* 0.039).This is shown in Table S 2.

### *Overall survival analysis

**Fig. 3:**
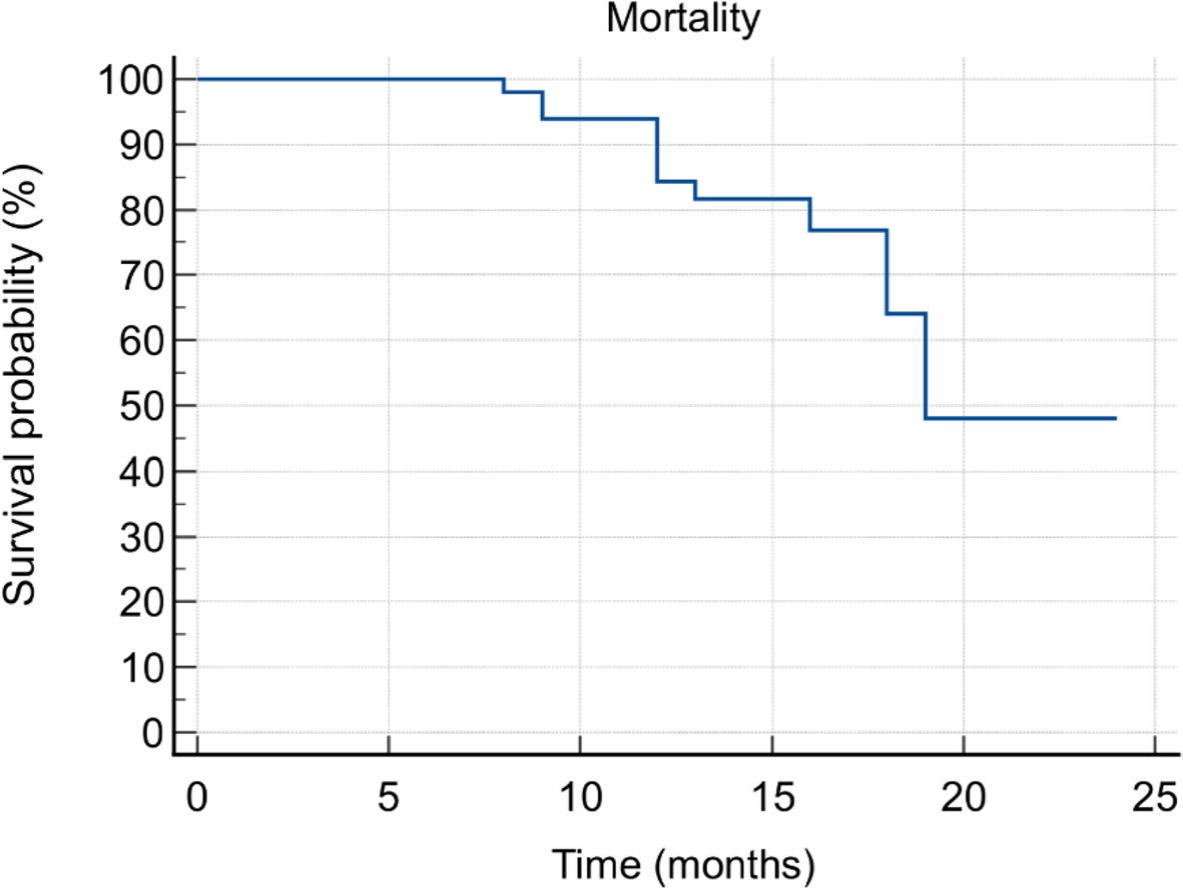
Kaplan Meier curve for OS analysis of patients

At the cutoff date (October 2024), 74% of the patients were alive and 26% had deceased. According to Kaplan–Meier curve analysis, the mean survival duration was 19.67 months (95% CI: 17.85 to 21.49), with a mortality rate of 26% (Table 5, Fig. 3).

**Table 5:** Overall survival analysis of the patients studied

**Item**	**N of events (%)**	**N censored (%)**			**Mean survival (95%CI)**	
**Total patients (*n* = 50)**	**13 (26%)**	**37 (74%)**			**19.67 (17.85 to 21.49)**	
**Item**	**N of events (%)**	**N censored (%)**	**Mean survival**		**HR (95%CI)**	**Log-rank** ***P*-value**
Complete response	0 (0%)	6 (100%)	24			
Partial response	3 (11.5%)	23 (88.5%)	19.11		Ref	**< 0.001**
Stable disease	2 (28.6%)	5 (71.4%)	18		2.33 (0.43 to 12.58)	
Progressive disease	8 (72.7%)	3 (27.3%)	12.57		9.9 (1.77 to 55.5)

There was a statistically significant association between response to treatment and overall survival according to log-rank analysis (*P <* 0.001). Mortality rates were 0% among patients who achieved complete response, 11.5% among those with partial response, 28.6% among those with stable disease, and 72.7% among with progressive disease. The hazard was higher in the stable disease and progressive disease groups than in the partial response group, with HR (95% CI) of 2.33 (0.43 to 12.58) and 9.9 (1.77 to 55.5), respectively (Figure S 2).

In univariate Cox regression analysis, pathology results and ECOG performance status were significantly associated with OS. Patients with squamous cell carcinoma and adenocarcinoma had significantly lower hazards of mortality than those with undifferentiated large cell NSCLC, with HR (95% CI) of 0.2 (0.05 to 0.74, *P =* 0.016) and 0.1 (0.02 to 0.51, *P =* 0.006), respectively. Compared with patients with ECOG PS1, those with PS2 showed a higher hazard of mortality (HR = 13.3, 95% CI: 1.73 to 102.59, *P =* 0.013).

After adjustment for the included factors, patients with ECOG PS2 had significantly higher hazards of mortality than those with PS1 (HR = 23.44, 95% CI: 1.39 to 396.06, *P =* 0.029) (Table S 3).

### *QoL assessment

EORTC QLQ-C30 assessment before treatment showed that the highest frequency was for having trouble doing strenuous activities, like carrying a heavy shopping bag or suitcase, (mean 3.62 ± 0.64), followed by trouble taking a long walk (3.54 ± 0.5), feeling nauseated (3.4 ± 0.95), being limited in pursuing hobbies or other leisure-time activities (3.3 ± 0.61), feeling depressed (3.26 ± 0.78), lacking appetite (3.12 ± 0.66), having social activities interfered with by their physical condition or medical treatment (3.04 ± 0.88), and being limited in doing work or other daily activities (3 ± 0.45). The mean overall health score was 3.34 ± 0.75, and the mean overall quality of life score was 3.38 ± 0.75 (Table S 4).

After 6 months of OV, the statements with the highest frequency by patients were having trouble doing strenuous activities (mean 3.36 ± 0.66), having trouble taking a long walk (3.24 ± 0.74), feeling tired (2.96 ± 0.7), needing rest (2.56 ± 0.86),feeling nauseated (2.56 ± 0.73), and having family life interfered with by their physical condition or medical treatment (2.54 ± 0.71). The mean overall health score was 4.76 ± 0.92, and the mean overall quality of life score was 4.84 ± 0.89 (Table S 5).

Comparison between pre- and post-treatment scores showed improvement in functioning and quality of life of patients. The functional impairment score was significantly decreased after treatment compared with before treatment (62.6 ± 12.85 vs. 76.64 ± 10.83, *P <* 0.001), and both overall health and QoL scores were significantly increased (*P <* 0.001), with a mean of 4.76 ± 0.92 vs. 3.34 ± 0.75 for the first and 4.84 ± 0.89 vs. 3.38 ± 0.75 for the latter (Table S 6).

In univariate linear regression analysis, tumor type according to pathology, stage, ECOG PS, receipt of concomitant chemoradiotherapy, and treatment duration were significantly associated with the functional impairment score of patients post-treatment. Patients with squamous cell carcinoma and adenocarcinoma showed significantly lower scores than those with undifferentiated large cell NSCLC, respectively, with coefficients (95% CI) of −13.28 (−23.33 to −3.24, *P =* 0.011) and −13.58 (−23.76 to −3.39, *P =* 0.01). Compared with patients with stage 3B tumors, those with stage 4B tumors showed significantly higher scores (coefficient = 12.07, 95% CI: 1.26 to 22.89, *P =* 0.029). Further, patients with ECOG PS2 had significantly higher scores than those with PS1 (coefficient = 7.39, 95% CI: 0.3 to 14.48, *P =* 0.041), and patients receiving concomitant chemoradiotherapy had lower scores than those not receiving it (coefficient = −7.96, 95% CI: −14.98 to −0.95, *P =* 0.027). Each 1-unit increase in treatment duration was significantly associated with a decrease in the score of −1.26 (95% CI: −2.23 to −0.29, *P =* 0.012).

In multiple analyses, we noted that patients within the age range of 65–69 years had significantly higher scores than those within the range of 60–64 years, with a coefficient of 10.66 (95% CI: 0.25 to 21.06, *P =* 0.045). Additionally, patients with squamous cell carcinoma and adenocarcinoma showed significantly lower scores than those with undifferentiated large cell NSCLC, respectively, with coefficients (95% CI) of −15.62 (−30.21 to −1.03, *P =* 0.037) and −15.79 (−28.29 to −3.29, *P =* 0.015). Patients receiving PD-L1 inhibitors had a significantly lower score than those not receiving them (coefficient = −9.67, 95% CI: −18.75 to −0.6, *P =* 0.037). Each 1-unit increase in treatment duration was associated with a decrease in the score of −1.66 (95% CI: −2.81 to −0.5, *P =* 0.006) (Table 6).

**Table 6:** Multivariable Model 3: Linear Regression for QoL Functional Impairment Score

Variable	Univariate analysis		Multivariable analysis	
	**Beta (95% CI)**	***p***>**-value**	**Beta (95% CI)**	***p***>**-value**
Histological Subtype (ref: Undifferentiated large cell)				
Squamous cell carcinoma	−13.28 (−23.33, −3.24)	**0.011**	−12.29 (−22.12, −2.47)	**0.015**
Adenocarcinoma	−13.58 (−23.76, −3.39)	**0.010**	−12.04 (−21.79, −2.29)	**0.017**
Tumor Stage (ref: 3B)				
3C	3.38 (−6.90, 13.65)	0.512	−2.75 (−12.74, 7.24)	0.582
4A	−0.59 (−9.76, 8.58)	0.897	−4.61 (−13.23, 4.02)	0.287
4B	12.07 (1.26, 22.89)	**0.029**	3.35 (−7.64, 14.34)	0.542
ECOG Performance Status (ref: PS1)				
PS2	7.39 (0.30, 14.48)	**0.041**	4.18 (−2.60, 10.95)	0.220
**Treatment duration (months)**	−1.26 (−2.23, −0.29)	**0.012**	−1.20 (−2.21, −0.18)	**0.022**

*CI* Confidence interval, Statistical significance at *P*-value < 0.05

## Discussion

Non-small-cell lung cancer (NSCLC) is the most commonly diagnosed cancer and the leading cause of death among males globally [21]. Platinum doublets with immunotherapy, or immunotherapy alone, are the standard palliative therapies for advanced disease; however not all patients are suitable candidates [22–24]. The metronomic concept was initially introduced more than 20 years ago, but substantial improvement has been observed in various solid tumors over the previous decade [9, 25].

The current study evaluated the treatment outcomes of 50 advanced/metastatic NSCLC patients who received a metronomic OV regimen three times per week until progression, patient refusal, intolerable toxicity, or death, as per the authorized indication.

In recent years, the metronomic formulation of vinorelbine has been investigated in several phase II NSCLC trials, notably in fragile patients such as the elderly and the infirm. Camerini et al. (2015) reported one of the first clinical trials with this innovative oral formulation: 43 chemo-naive elderly patients were treated with metronomic vinorelbine at a dosage of 50 mg three times a week, continuously. This therapy resulted in a 58.1% clinical benefit (PR + SD ≥ 6 weeks) with an 18% response rate, a median time-to-progression of 5 months, and an overall survival of 9 months. The therapy was well tolerated, with few significant adverse events [11]. Mencoboni et al. published a phase II study in 66 older patients in 2017, revealing a clinical benefit in 50% of patients with mild toxicity [26]. Other phase II trials confirmed the effectiveness and safety of vinorelbine's metronomic formulation in elderly and unfit patients [27, 28].

Our findings show that this study comprised older patients with locally advanced or metastatic non-small cell lung cancer, 36% were aged between 60 and 64, and 42% were aged between 65 and 69, with a male predominance of 78%. Pathology findings revealed squamous cell carcinoma, adenocarcinoma, and undifferentiated large cell NSCLC in 44%, 40%, and 16% of patients, respectively. Stage 3B tumors were the most prevalent (48%), followed by stage 4 A tumors (22%).

We acknowledge that defining the elderly population as individuals older than 60 years is lower than standard Western oncology thresholds, which typically begin at 65 or 70 years. However, this threshold was intentionally selected to reflect local demographic and national health realities. According to official data from the Central Agency for Public Mobilization and Statistics (CAPMAS), the average life expectancy at birth in Egypt is approximately 69.7 to 74.3 years. Furthermore, CAPMAS officially designates individuals aged 60 years and over as the elderly demographic segment within national public health assessments, noting that they comprise roughly 6.7% of the total population. Therefore, utilizing a 60-year cut-off ensures that our study generates highly representative, real-world clinical data tailored directly to the aging population within our specific regional context.

Furthermore, 46% exhibited ECOG PS1, whereas 54% had PS2. These findings are consistent with previous reports. For example, 30 patients with metastatic NSCLC who had progressed after first-line chemotherapy and subsequent immunotherapy were administered oral VNR on a metronomic schedule at eight medical oncology institutions in Sicily. In summary, there were 23 men (77%) and 7 women (23%), with a median age of 69 years (range 49–80) and a median ECOG performance level of 1. Seventy percent of the patients had adenocarcinoma, whereas 27% had squamous cell carcinoma [29].

In our study, the most prevalent response reported by patients was partial response (52%), followed by progressive disease (22%), stable disease (14%), and complete response (12%). Previously, researchers reported that the primary aim was attained, with 32% of patients achieving clinical improvement, which was excellent considering the patients' median age (79 years) and percentage of those with ECOG 2 (36%).

In terms of adverse events, 72% of the population experienced easy fatigability, 64% experienced decreased appetite, 60% experienced grade 1 and 2 anemia, 50% developed neutropenia (grades 1 and 2 in 36% and grade 3 in 14%), 50% experienced abdominal pain/spasm, 48% experienced arthralgia, 32% had constipation, 32% had renal impairment, and 30% developed neuropathy. Notably, 34% of patients stopped treatment due to progression.

This pattern of outcomes is similar to Gebbia V's earlier literature, which found that grade 3 toxicities were unusual, with two patients experiencing anemia requiring blood transfusions and one patient exhibiting temporary grade 3 neutropenia. Grade 1–2 fatigue and diarrhea were reported in eight (27%) and seven (23%) cases, respectively. Five individuals were found to have grade 1 neutropenia and did not require additional treatment. Four patients (13%) reported grade 1 constipation. Oral VNR was delayed by at least one week in nine individuals (30%) [29].

Other investigations found that toxicity was manageable, with just two patients (8%) experiencing grade 3 or 4 toxicity, corroborating Mencoboni et al.'s [26] results that metronomic vinorelbine had a better safety profile than the standard regimen.

In 2019, a meta-analysis of metronomic oral vinorelbine was published [30], including nine trials in both first- and second-line treatment. The median progression-free survival was 4.2 months, the median overall survival was 8.7 months, and 15.8% of patients developed grade 3–4 toxicity. The reviewers concluded that metronomic OV is an active and well-tolerated single-drug chemotherapy treatment in metastatic NSCLC and a reasonable therapy for frail patients.

Metronomic administration of chemotherapy leads to a cytostatic action, successfully shifting the treatment target from the rapidly mutating cancer cells to the more genetically stable tumor angiogenesis. Shifting the therapeutic target from tumor cells to tumor vasculature actively inhibits tumor regrowth between treatment cycles. This mechanism is supported by preclinical in vitro and in vivo models demonstrating that continuous, low-dose exposure to vinorelbine selectively induces apoptosis in activated endothelial cells and downregulates pro-angiogenic factors like VEGF, thereby sustaining prolonged disease stabilization even in heavily pretreated populations [31]

Based on our findings and the most current literature, we agree with this conclusion and believe metronomic vinorelbine is an excellent treatment option for patients with locally advanced and metastatic NSCLC without oncogenic drivers.

The findings of this study provide supportive evidence that patients had a mean progression-free duration of 13.74 months, with a progression rate of 34%. The mean survival time was 19.67 months, with 74% alive and 26% deceased. Previous studies reported that oral metronomic VNR at a dose of 50 mg/day three times per week was utilized as first-line treatment in elderly and/or unfit individuals, with encouraging results.

Camerini et al. [11] reported on a cohort of 43 patients who achieved an 18.6% overall response rate and a 58.1% disease control rate, with a median time to progression of 5 months (range 2–21) and a median overall survival of 9 months (range 3–29), with few grade 3 adverse effects. Another study of 66 patients treated with the same VNR regimen yielded similar findings [11].

Banna et al. employed first-line metronomic oral VNR at a dosage of 30 mg/day in a sample of 50 elderly/unfit patients. The overall disease control rate was 32%, 44%, and 26% in the first and subsequent lines, respectively. The median OS and PFS were 7.3 and 2.7 months, respectively. The most plausible reason for the current results may be the use of different dosing schedules and the absence of early treatment discontinuation [28].

Fernanda Estevinho et al. (2020) conducted a retrospective research study on 293 elderly NSCLC patients who were not eligible for conventional chemotherapy or tyrosine kinase inhibitors and were given oral metronomic VNR regardless of treatment line or dose. The study collected data from 19 Portuguese oncology centers between 2016 and 2018. The median age was 76 (39–94 years). The overall response rate was 18%, including 42 (18%) partial replies and zero complete responses. Overall, 54% of patients had stable disease, whereas 28% had progressive disease. The disease control rate was 72%. 21% of individuals had grade 3/4 toxicity. The results of this study are consistent with what we reported: metronomic scheduling is a meaningful and safe technique for treating advanced NSCLC patients [16].

Camerini A et al. (2021) conducted a multicenter, prospective, randomized, open-label phase II study on treatment-naive patients with TNM stage IIIB/IV NSCLC. Patients were given mVNR at a fixed dose of 50 mg × 3 or on a weekly regimen of 60–80 mg/m^2^ until disease progression or intolerable toxicity occurred. A total of 167 individuals were enrolled, with 83 and 84 in the mVNR and conventional arms, respectively. No significance difference in median OS was found. QoL was similar between arms. Metronomic oral Vinorelbine was associated with a significant reduction in toxicity, which is consistent with the results of our research. The EORTC QLQ C30 questionnaire showed similar changes from baseline in both therapy groups, with no deterioration in QoL scores [32].

In our study, we found that the comparison between pre- and post-treatment scores showed improvement in functional status and quality of life of patients, as the functional impairment score was significantly decreased after treatment compared to before, and both overall health and QoL scores were significantly increased with a statistically significant P-values.

## Conclusion

Metronomic OV represents a valuable therapeutic option for older individuals with locally advanced and metastatic NSCLC, with high efficacy, an acceptable safety profile, and improved patient QoL.

### Strengths and limitations

This study demonstrates several notable strengths. It achieved a 100% follow-up rate, thereby eliminating attrition bias and enhancing the internal validity of the findings. Additionally, the use of a prospectively maintained database ensured that QoL assessments were systematically collected according to a standardized protocol, minimizing recall bias and enhancing data reliability.

Nevertheless, certain limitations should be acknowledged. Although data collection was prospective, the analysis itself was conducted retrospectively, which may introduce inherent methodological constraints. Furthermore, the single-center design and small sample size (*n* = 50) may restrict the external validity and generalizability of the findings to broader populations.

Additionally, granular smoking history metrics, such as the comprehensive Brinkman Index, were not systematically captured for all patients, which precluded a secondary correlation analysis with treatment-related toxicities.

## Supplementary Information

Supplementary Material 1.

Supplementary Material 2.

## Data Availability

The data supporting the conclusions of this study are available from the corresponding author upon reasonable request.

## Abbreviations

AE: Adverse events
CDC: Center for Disease Control and Prevention
CR: Complete response
CT: Computed Tomography
DM: Diabetes Mellitus
ECOG: Eastern Cooperative Oncology Group
FOMSCU: Faculty of Medicine Suez Canal University
HTN: Hypertension
IQR: Interquartile range
MC: Metronomic chemotherapy
MRI: Magnetic Resonance Imaging
NSCLC: Non-small cell lung cancer
ORR: Overall response rate
OS: Overall survival
OV: Oral vinorelbine
PFS: Progression-free survival
PR: Partial response
PS: Performance status
QoL: Quality of Life
RECIST: Response evaluation criteria in solid tumors
SAEs: Serious Adverse Events
SCUH: Suez Canal University Hospital
SD: Stable Disease
