# Supplementary material for: Efficacy and safety of metronomic oral vinorelbine in elderly patients with locally advanced and metastatic non-small cell lung cancer
Source: BMC Cancer. 2026 Jul 17;26:885. doi: 10.1186/s12885-026-16517-x (PMC13393617; doi:10.1186/s12885-026-16517-x)
Supplement: Supplementary file 1 — Supplementary Material 1. [file 12885_2026_16517_MOESM1_ESM.docx]

Table S 1: Response of the studied patients (n=50)

| Item | N | % |
| --- | --- | --- |
| Complete response | 6 | 12.0 |
| Partial response | 26 | 52.0 |
| Stationary coarse | 7 | 14.0 |
| Progressive disease | 11 | 22.0 |

Table S 2: Cox-regression analysis for factors associated with PFS of patients

| Item | Univariate analysis | | | Multivariable analysis | | |
| --- | --- | --- | --- | --- | --- | --- |
|  | **HR** | **95%CI** | **P-value** | **HR** | **95%CI** | **P-value** |
| Age group (years) |  |  |  |  |  |  |
| 60 – 64 | Ref |  |  | Ref |  |  |
| 65 – 69 | 1.41 | 0.51 to 3.87 | 0.509 | 7.49 | 0.65 to 86.8 | 0.107 |
| ≥70 | 0.24 | 0.03 to 1.99 | 0.186 | 2.52 | 0.05 to 117.06 | 0.637 |
| Sex |  |  |  |  |  |  |
| Male | Ref |  |  | Ref |  |  |
| Female | 0.49 | 0.11 to 2.15 | 0.344 | 1.18 | 0.13 to 10.33 | 0.881 |
| Comorbidities (+ve) | 1.09 | 0.42 to 2.88 | 0.855 | 1.22 | 0.25 to 5.87 | 0.803 |
| Pathology |  |  |  |  |  |  |
| Undifferentiated large cell NSCLC | Ref |  |  | Ref |  |  |
| Squamous cell carcinoma | 0.49 | 0.16 to 1.48 | 0.205 | 0.08 | 0 to 2.75 | 0.164 |
| Adenocarcinoma | 0.28 | 0.08 to 1.01 | 0.052 | 0.19 | 0.02 to 1.95 | 0.161 |
| Stage |  |  |  |  |  |  |
| 3B | Ref |  |  | Ref |  |  |
| 3C | 3.47 | 0.73 to 16.52 | 0.118 | 6.33 | 0.63 to 64.2 | 0.118 |
| 4A | 3.09 | 0.82 to 11.55 | 0.094 | 10.25 | 0.52 to 200.65 | 0.125 |
| 4B | 6.53 | 1.63 to 26.08 | **0.008** | 20.31 | 1.46 to 282.04 | **0.025** |
| ECOG |  |  |  |  |  |  |
| PS1 | Ref |  |  | Ref |  |  |
| PS2 | 3 | 0.97 to 9.2 | 0.055 | 8.42 | 1.11 to 63.81 | **0.039** |
| 1st line chemotherapy (received) | 1.53 | 0.54 to 4.38 | 0.425 | 6.34 | 0.88 to 45.4 | 0.066 |
| Concomitant chemoradiotherapy over lung mass (received) | 0.2 | 0.06 to 0.7 | **0.012** | 4.2 | 0.16 to 107.34 | 0.385 |
| PDL1 (+ve) | 0.7 | 0.2 to 2.45 | 0.581 | 0.55 | 0.05 to 5.49 | 0.61 |

HR: Hazard ratio, CI: Confidence interval, Statistical significance at P value<0.05

Table S 3: Cox-regression analysis for factors associated with OS of patients

| Item | Univariate analysis | | | Multivariable analysis | | |
| --- | --- | --- | --- | --- | --- | --- |
|  | **HR** | **95%CI** | **P-value** | **HR** | **95%CI** | **P-value** |
| Age group (years) |  |  |  |  |  |  |
| 60 – 64 | Ref |  |  | Ref |  |  |
| 65 – 69 | 0.86 | 0.27 to 2.76 | 0.806 | 0.56 | 0.06 to 5.13 | 0.605 |
| ≥70 | 0.25 | 0.03 to 2.19 | 0.212 | 0.45 | 0.01 to 23.57 | 0.691 |
| Sex |  |  |  |  |  |  |
| Male | Ref |  |  | Ref |  |  |
| Female | 0.3 | 0.04 to 2.31 | 0.248 | 3.31 | 0.11 to 98.5 | 0.489 |
| Smoking (+ve) | 2.65 | 0.78 to 9 | 0.119 | 3.89 | 0.28 to 54.97 | 0.314 |
| Comorbidities (+ve) | 1.02 | 0.33 to 3.16 | 0.975 | 1.33 | 0.14 to 12.33 | 0.804 |
| Pathology |  |  |  |  |  |  |
| Undifferentiated large cell NSCLC | Ref |  |  | Ref |  |  |
| Squamous cell carcinoma | 0.2 | 0.05 to 0.74 | **0.016** | 0.65 | 0.05 to 8.72 | 0.743 |
| Adenocarcinoma | 0.1 | 0.02 to 0.51 | **0.006** | 0.12 | 0.01 to 1.52 | 0.103 |
| ECOG |  |  |  |  |  |  |
| PS1 | Ref |  |  | Ref |  |  |
| PS2 | 13.3 | 1.73 to 102.59 | **0.013** | 23.44 | 1.39 to 396.06 | **0.029** |
| 1st line chemotherapy (received) | 0.6 | 0.2 to 1.85 | 0.376 | 0.7 | 0.08 to 6.37 | 0.753 |
| PDL1 (+ve) | 1.1 | 0.3 to 4.01 | 0.886 | 0.94 | 0.03 to 27.61 | 0.974 |

HR: Hazard ratio, CI: Confidence interval, Statistical significance at P value<0.05

Table S 4: EORTC QLQ-C30 assessment of patient pre-treatment

| **Item** | **Not at all** | | **A little** | **Quite a bit** | | **Very much** | | **Score**  **(mean ± SD)** |
| --- | --- | --- | --- | --- | --- | --- | --- | --- |
| Do you have any trouble doing strenuous activities, like carrying a heavy shopping bag or a suitcase? | 0 (0%) | | 4 (8%) | 11 (22%) | | 35 (70%) | | 3.62 ± 0.64 |
| Do you have any trouble taking a long walk? | 0 (0%) | | 0 (0%) | 23 (46%) | | 27 (54%) | | 3.54 ± 0.5 |
| Do you have any trouble taking a short walk outside of the house? | 4 (8%) | | 14 (28%) | 32 (64%) | | 0 (0%) | | 2.56 ± 0.64 |
| Do you need to stay in bed or a chair during the day? | 0 (0%) | | 13 (26%) | 32 (64%) | | 5 (10%) | | 2.84 ± 0.58 |
| Do you need help with eating, dressing, washing yourself or using the toilet? | 8 (16%) | | 22 (44%) | 20 (40%) | | 0 (0%) | | 2.24 ± 0.72 |
| Were you limited in doing either your work or other daily activities? | 0 (0%) | | 5 (10%) | 40 (80%) | | 5 (10%) | | 3 ± 0.45 |
| Were you limited in pursuing your hobbies or other leisure time activities? | 0 (0%) | | 4 (8%) | 27 (54%) | | 19 (38%) | | 3.3 ± 0.61 |
| Were you short of breath? | 5 (10%) | | 11 (22%) | 32 (64%) | | 2 (4%) | | 2.62 ± 0.73 |
| Have you had pain? | 10 (20%) | | 27 (54%) | 13 (26%) | | 0 (0%) | | 2.06 ± 0.68 |
| Did you need to rest? | 0 (0%) | | 12 (24%) | 29 (58%) | | 9 (18%) | | 2.94 ± 0.65 |
| Have you had trouble sleeping? | 7 (14%) | | 17 (34%) | 19 (38%) | | 7 (14%) | | 2.52 ± 0.91 |
| Have you felt weak? | 2 (4%) | | 13 (26%) | 25 (50%) | | 10 (20%) | | 2.86 ± 0.78 |
| Have you lacked appetite? | 0 (0%) | | 8 (16%) | 28 (56%) | | 14 (28%) | | 3.12 ± 0.66 |
| Have you felt nauseated? | 5 (10%) | | 1 (2%) | 13 (26%) | | 31 (62%) | | 3.4 ± 0.95 |
| Have you vomited? | 5 (10%) | | 12 (24%) | 32 (64%) | | 1 (2%) | | 2.58 ± 0.7 |
| Have you been constipated? | 7 (14%) | | 21 (42%) | 22 (44%) | | 0 (0%) | | 2.3 ± 0.71 |
| Have you had diarrhea? | 23 (46%) | | 24 (48%) | 3 (6%) | | 0 (0%) | | 1.6 ± 0.61 |
| Were you tired? | 0 (0%) | | 18 (36%) | 23 (46%) | | 9 (18%) | | 2.82 ± 0.72 |
| Did pain interfere with your daily activities? | 4 (8%) | | 18 (36%) | 21 (42%) | | 7 (14%) | | 2.62 ± 0.83 |
| Have you had difficulty concentrating on things, like reading a newspaper or watching television? | 2 (4%) | | 22 (44%) | 19 (38%) | | 7 (14%) | | 2.62 ± 0.78 |
| Did you feel tense? | 25 (50%) | | 16 (32%) | 4 (8%) | | 5 (10%) | | 1.78 ± 0.97 |
| Did you worry? | 6 (12%) | | 17 (34%) | 14 (28%) | | 13 (26%) | | 2.68 ± 1 |
| Did you feel irritable? | 4 (8%) | | 17 (34%) | 16 (32%) | | 13 (26%) | | 2.76 ± 0.94 |
| Did you feel depressed? | 1 (2%) | | 7 (14%) | 20 (40%) | | 22 (44%) | | 3.26 ± 0.78 |
| Have you had difficulty remembering things? | 3 (6%) | | 17 (34%) | 27 (54%) | | 3 (6%) | | 2.6 ± 0.7 |
| Has your physical condition or medical treatment interfered with your family life? | 6 (12%) | | 14 (28%) | 17 (34%) | | 13 (26%) | | 2.74 ± 0.99 |
| Has your physical condition or medical treatment interfered with your social activities? | 2 (4%) | | 12 (24%) | 18 (36%) | | 18 (36%) | | 3.04 ± 0.88 |
| Has your physical condition or medical treatment caused you financial difficulties? | 7 (14%) | | 12 (24%) | 24 (48%) | | 7 (14%) | | 2.62 ± 0.9 |
|  | **1** | **2** | **3** | **4** | **5** | **6** | **7** | **Score**  **(mean ± SD)** |
| How would you rate your overall health during the past week? | 0 (0%) | 4 (8%) | 29 (58%) | 13 (26%) | 4 (8%) | 0 (0%) | 0 (0%) | 3.34 ± 0.75 |
| How would you rate your overall quality of life during the past week? | 0 (0%) | 4 (8%) | 27 (54%) | 15 (30%) | 4 (8%) | 0 (0%) | 0 (0%) | 3.38 ± 0.75 |

Categorical data are presented as frequency (%)

Table S 5: EORTC QLQ-C30 assessment of patients after 6 months OV

| **Item** | **Not at all** | | **A little** | **Quite a bit** | | **Very much** | | **Score**  **(mean ± SD)** |
| --- | --- | --- | --- | --- | --- | --- | --- | --- |
| Do you have any trouble doing strenuous activities, like carrying a heavy shopping bag or a suitcase? | 0 (0%) | | 5 (10%) | 22 (44%) | | 23 (46%) | | 3.36 ± 0.66 |
| Do you have any trouble taking a long walk? | 0 (0%) | | 9 (18%) | 20 (40%) | | 21 (42%) | | 3.24 ± 0.74 |
| Do you have any trouble taking a short walk outside of the house? | 4 (8%) | | 37 (74%) | 5 (10%) | | 4 (8%) | | 2.18 ± 0.69 |
| Do you need to stay in bed or a chair during the day? | 10 (20%) | | 33 (66%) | 3 (6%) | | 4 (8%) | | 2.02 ± 0.77 |
| Do you need help with eating, dressing, washing yourself or using the toilet? | 31 (62%) | | 15 (30%) | 3 (6%) | | 1 (2%) | | 1.48 ± 0.71 |
| Were you limited in doing either your work or other daily activities? | 3 (6%) | | 31 (62%) | 10 (20%) | | 6 (12%) | | 2.38 ± 0.78 |
| Were you limited in pursuing your hobbies or other leisure time activities? | 0 (0%) | | 34 (68%) | 16 (32%) | | 0 (0%) | | 2.32 ± 0.47 |
| Were you short of breath? | 11 (22%) | | 27 (54%) | 1 (2%) | | 11 (22%) | | 2.24 ± 1.04 |
| Have you had pain? | 18 (36%) | | 26 (52%) | 1 (2%) | | 5 (10%) | | 1.86 ± 0.88 |
| Did you need to rest? | 3 (6%) | | 25 (50%) | 13 (26%) | | 9 (18%) | | 2.56 ± 0.86 |
| Have you had trouble sleeping? | 11 (22%) | | 22 (44%) | 13 (26%) | | 4 (8%) | | 2.2 ± 0.88 |
| Have you felt weak? | 6 (12%) | | 30 (60%) | 8 (16%) | | 6 (12%) | | 2.28 ± 0.83 |
| Have you lacked appetite? | 1 (2%) | | 31 (62%) | 14 (28%) | | 4 (8%) | | 2.42 ± 0.67 |
| Have you felt nauseated? | 1 (2%) | | 26 (52%) | 17 (34%) | | 6 (12%) | | 2.56 ± 0.73 |
| Have you vomited? | 24 (48%) | | 25 (50%) | 1 (2%) | | 0 (0%) | | 1.54 ± 0.54 |
| Have you been constipated? | 16 (32%) | | 24 (48%) | 10 (20%) | | 0 (0%) | | 1.88 ± 0.72 |
| Have you had diarrhea? | 46 (92%) | | 0 (0%) | 4 (8%) | | 0 (0%) | | 1.16 ± 0.55 |
| Were you tired? | 0 (0%) | | 13 (26%) | 26 (52%) | | 11 (22%) | | 2.96 ± 0.7 |
| Did pain interfere with your daily activities? | 21 (42%) | | 15 (30%) | 14 (28%) | | 0 (0%) | | 1.86 ± 0.83 |
| Have you had difficulty concentrating on things, like reading a newspaper or watching television? | 5 (10%) | | 36 (72%) | 4 (8%) | | 5 (10%) | | 2.18 ± 0.75 |
| Did you feel tense? | 25 (50%) | | 18 (36%) | 7 (14%) | | 0 (0%) | | 1.64 ± 0.72 |
| Did you worry? | 12 (24%) | | 27 (54%) | 5 (10%) | | 6 (12%) | | 2.1 ± 0.91 |
| Did you feel irritable? | 9 (18%) | | 29 (58%) | 11 (22%) | | 1 (2%) | | 2.08 ± 0.7 |
| Did you feel depressed? | 10 (20%) | | 22 (44%) | 13 (26%) | | 5 (10%) | | 2.26 ± 0.9 |
| Have you had difficulty remembering things? | 7 (14%) | | 22 (44%) | 16 (32%) | | 5 (10%) | | 2.38 ± 0.85 |
| Has your physical condition or medical treatment interfered with your family life? | 0 (0%) | | 29 (58%) | 15 (30%) | | 6 (12%) | | 2.54 ± 0.71 |
| Has your physical condition or medical treatment interfered with your social activities? | 1 (2%) | | 28 (56%) | 16 (32%) | | 5 (10%) | | 2.5 ± 0.71 |
| Has your physical condition or medical treatment caused you financial difficulties? | 8 (16%) | | 22 (44%) | 11 (22%) | | 9 (18%) | | 2.42 ± 0.97 |
|  | **1** | **2** | **3** | **4** | **5** | **6** | **7** | **Score**  **(mean ± SD)** |
| How would you rate your overall health during the past week? | 0 (0%) | 1 (2%) | 4 (8%) | 10 (20%) | 26 (52%) | 9 (18%) | 0 (0%) | 4.76 ± 0.92 |
| How would you rate your overall quality of life during the past week? | 0 (0%) | 1 (2%) | 4 (8%) | 6 (12%) | 30 (60%) | 9 (18%) | 0 (0%) | 4.84 ± 0.89 |

Categorical data are presented as frequency (%)

Table S 6: Total EORTC QLQ-C30 scores of patients pre and post treatment

| **Item** | **Pre-treatment** | **Post-treatment** | **P-value** |
| --- | --- | --- | --- |
| Functional impairment | 76.64 ± 10.83 | 62.6 ± 12.85 | **<0.001** |
| Overall health | 3.34 ± 0.75 | 4.76 ± 0.92 | **<0.001** |
| Overall QOL | 3.38 ± 0.75 | 4.84 ± 0.89 | **<0.001** |

Numerical data are presented as mean ± SD, Statistical significance at P-value < 0.05.
