# Supplementary material for: Efficacy and safety of metronomic oral vinorelbine in elderly patients with locally advanced and metastatic non-small cell lung cancer
Source: BMC Cancer. 2026 Jul 17;26:885. doi: 10.1186/s12885-026-16517-x (PMC13393617; doi:10.1186/s12885-026-16517-x)
Supplement: Supplementary file 2 — Supplementary Material 2. [file 12885_2026_16517_MOESM2_ESM.docx]

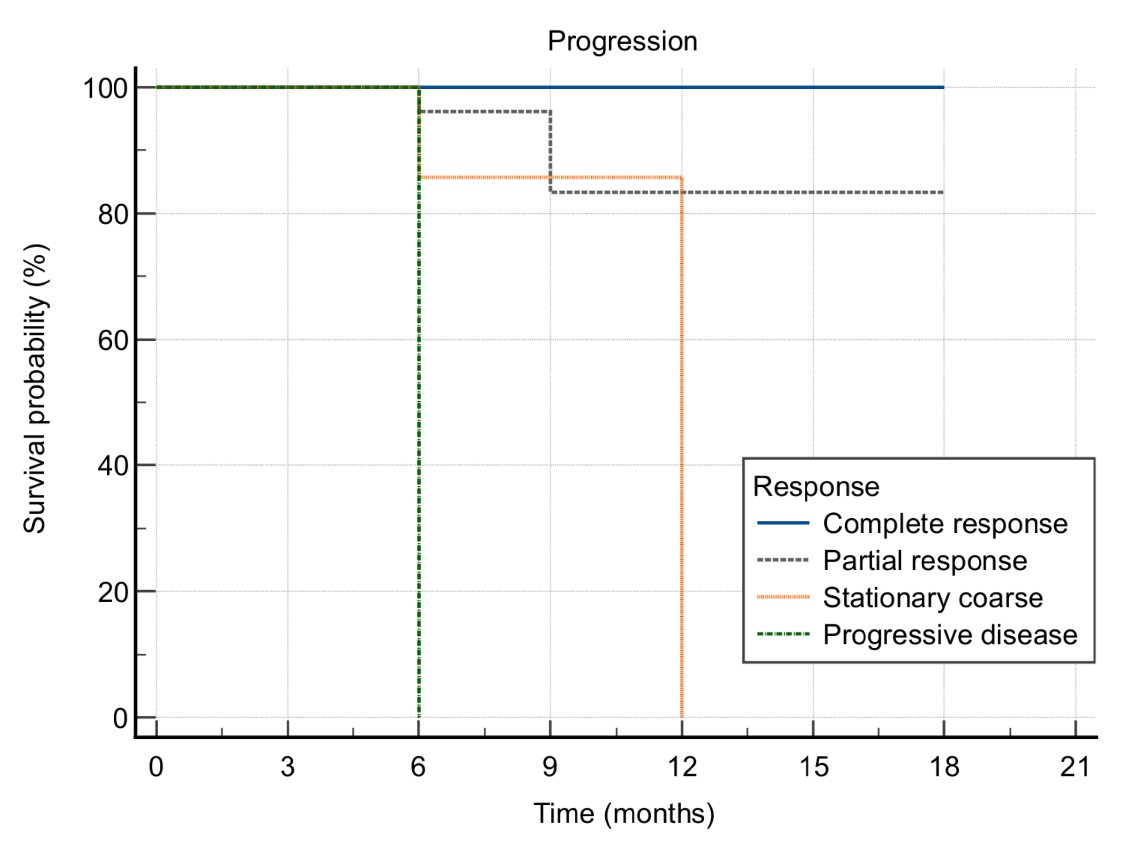


Figure S 1: Kaplan Meier curve for PFS analysis of patients according to response to treatment


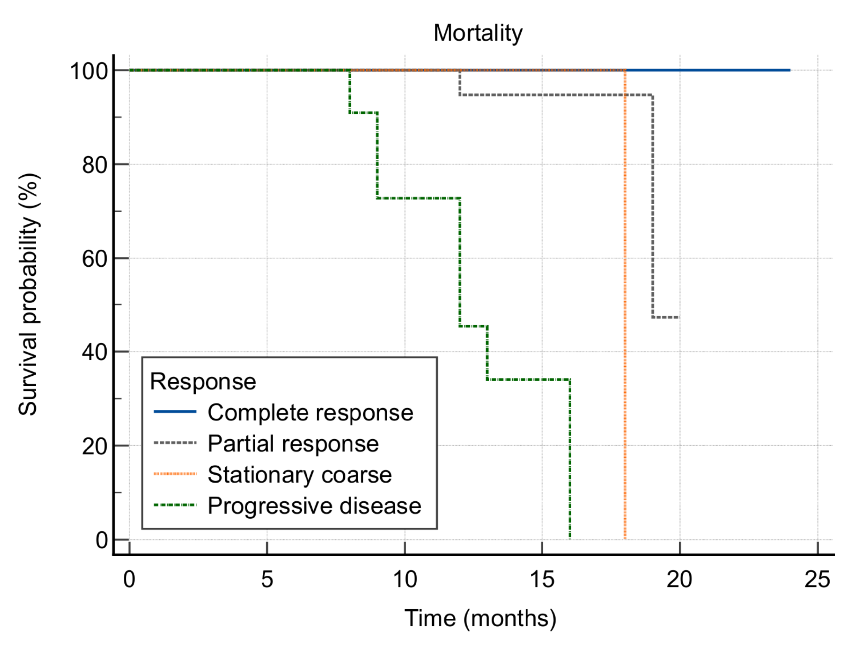


Figure S 2: Kaplan Meier curve for OS analysis of patients according to response to treatment
